# Naso-Pharyngeal Polypus, Attached to the Basilar Process of the Occipital, and Body of the Sphenoid Bones, Successfully Removed by a Section, Displacement, and Subsequent Re-Placement and Re-Union of the Superior Maxillary Bone

**Published:** 1867-12

**Authors:** 


### ARTICLE X.

*Naso-pharyngeal Polypus, attached to the Basilar Process of the Occipital, and body of the Sphenoid Bones, successfully removed by a Section, Displacement, and subsequent Re-placement and Re-union of the Superior Maxillary Bone.*

*City Hospital.*—Reported by Dr. CHEEVER.

THE patient, a student, eighteen years old, entered the Hospital, July 20th, with the following history:—

About two and a half years ago he had profuse epistaxis, which continued twenty-four hours. During the six months next following this, he had only occasional slight attacks. At the end of that period he found that his right nostril was wholly obstructed, and he has never since been able to blow through it. He soon became aware of a growth behind that nostril, which gradually but steadily increased, until within a few weeks of the time of entrance, when it grew rapidly. There was some discharge, but it was not offensive until quite recently.

At the time of admission to the Hospital, the soft palate was found to be depressed and pushed forward until it hung at a right angle with the hard palate, and both it and the tonsil were inflamed. At the right side of the fauces a small ulcerated patch could be seen. By the finger, a tumor could be felt behind the soft palate, firm, full and lobulated, and extending farther up than the finger could be carried. Its lower lobes hung down into the throat. The whole of the upper part of the pharnyx was occupied, except a small space on the left. Nothing could be passed into the pharynx through the right nostril, but the left was clear. Hearing was imperfect in the right ear; and the respiration was mostly through the mouth. The microscopical examination of the debris of the tumor, removed by a digital examination, revealed only blood corpuscles, pus cells and *fibrous* tissue. There was no evidence of malignancy.

The patient was able to take liquid or soft food only. The general health was good, with no hereditary predisposition. He was very desirous of an operation for the removal of the tumor.

There was no question that the tumor must be removed, or, before long, cause the death of the patient. The problem to be solved was as to the best method of operating. Three modes offered themselves for consideration.

1st. By ligature, or the *ecraseur*, through the nares. This was impracticable, because nothing could be passed through the right nostril opposite to which the bulk of the tumor lay; also because it was not a pedunculated growth. Were it practicable, it could only cut off the growth, without eradicating it; and it would, probably, speedily recur.

2d. By section of the soft palate, of the hard palate, and removal through the mouth, with a subsequent operation for staphyloraphy. This mode, advocated and revived by Nélaton, but really as old as Hippocrates, was abandoned on account of the size and high attachments of the tumor, and the fear that room enough to manipulate could not be got through the section of the hard palate.

3d. By removing the superior maxilla—a plain and easy way, but accompanied by deformity and serious mutilation. Here was a young man with a healthy jaw and perfect teeth, and the disease wholly behind it. Could a portion of the jaw be saved? or even the whole replaced? I decided to make a horizontal section of the jaw; depress it, saving all attachments of the soft parts possible; see if the tumor could thus be reached; and, if practicable, to replace the jaw, and try to save it.

July 23d.—*Operation.* The patient was etherized during the first part of the operation, and partial etherization was renewed at intervals; he was seated in a chair, with the head on a pillow.

An incision was made from just below the inner canthus of right eye, downwards by the side of the nose, following the naso-labial fissure, to the corner of the mouth. The inner flap was dissected up until the symphysis was exposed; and the outer, until nearly the whole of the superior maxilla was free. With a narrow bladed saw, about three inches long, the superior maxilla was now divided transversely, about half an inch below the floor of the orbit. The blade of the saw was plunged into the zygomatic fossa, and the front and back walls of the antrum were sawn through horizontally, starting just below the articulation with the malar bone, and terminating in the anterior nares, at the lower end of the nasal bone. The ala of the nose having been lifted up, the right central incisor was next extracted. Strong bone forceps were now used to divide the alveolar process, through the socket of the right central incisor. The cut included the *alveolus only.* The hard and soft palate were not touched. The bone was now held by the palate process, palate bone, and its co-ossification with the pterygoid processes. Seizing the alveolar process with strong tooth-forceps, the whole section of the superior maxilla was bent down and displaced into the mouth. The antrum was found to be filled by one lobe of the tumor without attachment, while the body of the tumor was attached to the upper, back part and right side of the pharynx and to the base of the sphenoid bone. The body was very firm, and the attachments were broad, covering a space two inches square. These, with considerable difficulty, were severed by scissors, introduced through the opening above the depressed section of the superior maxilla, and the base was cauterized repeatedly with strong nitric acid. The hæmorrhage, which was not excessive, was thereby effectually checked. Four ligatures were applied to bleeding vessels in the course of the first incisions. With the forefinger of the right hand in the throat, and the left in the cavity above the section of the maxilla, they could be made to meet freely, and explore thoroughly the pharnyx, which was now found entirely clear of obstruction.

The superior maxillary bone was now hanging with its antrum exposed ; and attached by the bent, or broken hard palate, the unbroken soft palate, and the broken osseous, and unbroken muscular and vascular attachments of the pterygoid process of the spenoid bone. On these attachments we were to rely for the restoration of the bone. The maxilla was easily pushed up into its place, and held by a silver wire passed round the left central, and right incisor teeth ; and by the closing of the lower jaw. The flaps of skin were accurately approximated, and united in place by six interrupted sutures.

At the close of the operation, the pulse was 120 and of fair strength. Wine, iced milk, beef tea and opium were ordered, *pro re nata.* 7 P.M. pulse 132. Reaction good. Takes nourishmsnt freely. No vomiting or pain. Urine free. ℞. Pulv. Doveri, grs. x. 10 P.M. Sleeping quictly. Pulse 88. Respiration free.

July 24th, A.M.—Pulse 120, good. Patient in good spirits. Face drawn a trifle to left side. There is little swelling of the face, and some offensive odor from the clotted blood. The parts syringed with ℞. Tr. myrrh., ℥ i.; aqua, ℥ iv. M. P.M. Pulse 130. No pain. Takes beef-tea, milk, eggs, &c., freely. Sleeps considerably. Functions regular.

July 25th.—Pulse 96. Except some drowsiness, feels very well. Appetite good. Sutures removed, and good union found. Eye nearly closed by adjacent swelling. The nares and pharynx to be syringed twice a day.

July 26th.—Pulse 112. Looks brighter. Eye very much better. Union of flaps quite firm.

July 28th.—Improving. Discharges more moderate and less offensive. Upper jaw in good position, having fallen only a very little. Appetite good ; bowels regular ; sleeps well ; no pain.

July 29th.—A small swelling of palate just behind incisor teeth lanced. Discharge of pure blood. General condition of patient as good as usual.

July 30th.—Ligatures all away. External wound entirely healed.

Aug. 1st.—Quite comfortable. Discharge diminishing. A small piece of gutta-percha was moulded between upper and lower jaw of right side, and a bandage around the head and chin to keep the bone up in place.

Aug. 3d.—Steadily improving. Jaw in good position.

Aug. 5th.—Discharge from right nostril about normal. Patient walks about Hospital grounds, without suffering any inconvenience or pain.

Aug. 12th.—Still doing admirably, *No purulent discharge.* Some pain on pressure where the jaw was sawed across. Plug between jaws continued. No appreciable motion of parts of bone.

Aug. 22d.—Progressing very favorably. Bandage and gutta-percha removed. Union of maxilla firm. Four weeks since operation.

Aug. 28th.—Discharged at his own request, well. Still wearing the wire about the teeth.

Sept. 2d.—Reported himself at the Hospital, in excellent condition. The wire removed. Union perfect; able to chew; now six weeks since the operation. Respiration clear. Examined with the rhinoscope by Dr. Langmaid, and the pharynx found healthy. A slight catarrh from the right nostril; nothing more.—*Boston Med. and Surg. Journal.*

